# Sperm impairing microbial factor: potential candidate for male contraception

**DOI:** 10.1186/s12958-020-00654-4

**Published:** 2020-09-30

**Authors:** Aditi Chauhan, Deepali Thaper, Vijay Prabha

**Affiliations:** Department of Microbiology, Panjab University, Chandigarh, 160014 India

**Keywords:** Sperm immobilization factor, Vas deferens, Azoospermia and contraception

## Abstract

**Background:**

Despite significant advances in contraceptive options for women, vasectomy and condoms are the only options available for male contraception. Due to this limitation, the burden of contraception resides on the shoulders of females only. Therefore, there is an urgent need to develop a safe, effective and reversible method of contraception for men. Amongst the alternative approaches, microbial derived products are gaining attention of the scientific world to combat unintended pregnancies. Earlier in our laboratory, sperm impairing microbial factor (Sperm immobilization factor) isolated from *Staphylococcus aureus* has shown excellent contraceptive efficacy in female mice. Keeping this in mind, the present study was carried out to exploit the sperm immobilization factor (SIF) as potential male contraceptive using vas deferens for administration in mouse model.

**Methods:**

SIF (10, 50, 100 or 200 μg) was inoculated in the lumen of right vas deferens whereas the left vas deferens served as control. The mice were sacrificed at Day 3, 7, 14, 21, 30, 45, 60 and 90 after inoculation and the results in terms of change in body weight, seminal parameters, Tissue somatic indices (TSI), haematological parameters, serum level of testosterone, lipid peroxidation and histology were studied. In order to ratify the SIF induced azoospermia SIF (200 μg) was administered with different doses viz. 100, 200, 300, 400 or 500 μg of SIF binding receptor extracted from mouse spermatozoa.

**Results:**

The weight profile studies of all the experimental groups showed no significant change in the initial and final body weight. In case of seminal parameters, the results revealed that right vas deferens treated with SIF showed azoospermia and with 200 μg of SIF it persisted up to 90 days. TSI of reproductive organs and non-reproductive organs showed no significant change in all the experimental groups. The haematological indices were found to be unaltered throughout the course of investigation however significant decrease in testosterone level was observed in the treated mice. The treatment also affected the oxidative status of the testis. Further, histological studies revealed hypospermatogenesis and late maturation arrest on treated side whereas the left side which served as control showed normal tissue histology. SIF induced azoospermia was ameliorated when administered with 400 μg of SIF binding receptor from mouse spermatozoa.

**Conclusion:**

SIF, when administered via intra vas deferens route, could lead to complete azoospermia. Therefore, it could be considered as a potential male contraceptive.

## Background

The alarming rise in the world population has left the nations fuming in rage, in terms of population density [1] and contraception has emerged as a key solution to curb this menace. Today most of the contraceptive options available in the market are inclined towards the females since it is the female, who bears the burden of pregnancy. Researchers have been relentlessly involved in creating new contraceptive options for men since antiquity. Still, condoms and vasectomy are the only methods of contraception which are most commonly used. For the development of new male contraceptives, the two main approaches that have contemplated thus far are hormonal and non-hormonal approaches [2]. However, the several long-term side effects of hormonal approach such as elevated blood pressure, local skin reactions, increased sweating at night and enlarged prostate in men limit their usage [3–5]. While in case of non-hormonal approach viz. herbal plants there are several concerns regarding their use such as little knowledge about quality, safety and efficacy of their preparations along with purification of the active ingredient. Besides, there are few therapeutic agents such as Miglustat, Indenopyridines, Eppin, Adjudin, and Gamendazole, which have the potential to be utilized as contraceptive agents but, severe effects of these chemical compounds ranging from hypokalemia, neurotoxicity to suppression of immune response and irreversibility have prevented their extrapolation from mice to humans [6].

The frustrating struggle to develop a male contraceptive has led to the exploration of alternative approaches which offer reversibility, localized action, safety with minimal side effects and greater efficacy. In this context, Lohiya et al. [7] have revolutionized the world of contraception by introducing a new approach wherein styrene-maleic anhydride (SMA) dissolved in dimethyl sulfoxide (DMSO) was administered through vas deferens. Being localized and more specific in action, it draws greater attention. Earlier, Upadhyay et al. [8] have also induced long term block of fertility in male mice by a single unilateral Intra vas administration of neem oil. So, this intrigued us to exploit vas deferens for administration of microbial factor isolated from *Staphylococcus aureus* as a contraceptive agent in male mice, which has shown sperm immobilization in vitro and admirable contraceptive efficacy in female mice [9].

## Materials and methods

### Experimental animals

Sexually mature male Balb/c mice (5–6 weeks old, 25 ± 2 g) were used in the present study and they were individually housed in polypropylene cages. The animals were fed with standard pellet food and water ad libitum and (12:12, dark: light cycle) standard laboratory conditions were retained. All the experimental work has been executed in consensus with the procedures approved by the Institutional Animal Ethics Committee, Panjab University vide letter no. PU/IAEC/S/16/140. All experiments were successfully completed in agreement with the guidelines of the Committee for the Purpose of Control and Supervision of Experiments on Animals (CPCSEA).

### Microorganism

A strain of *S. aureus* causing sperm immobilization in vitro*,* isolated previously in our laboratory was used in the present study [9].

### Isolation and purification of sperm immobilization factor (SIF) from *S. aureus*

The SIF was isolated and purified from 72 h old cell culture of *S. aureus* by the method earlier standardized in the laboratory [9].

### Intravasal inoculation procedure

Under surgical conditions, male Balb/c mice were inoculated with different concentrations of SIF viz. 10, 50, 100 and 200 μg in the right vas deferens under anaesthesia of ketamine and xylazine and for each dose, 3 mice were inoculated. Following a scrotal incision in the inguinal region, the right testis, vas deferens and epididymis were exteriorized. The inoculum (20 μl) was instilled into the lumen of the right vas deferens by using a 27-gauge needle towards the direction of the epididymis. The incision was closed with 3–0 silk suture. A preliminary study was carried out before starting this study, in which male mice were surgically administered with single dose of 20 μl PBS in the right vas deferens and the results showed that both the TSI and the seminal parameters of the treated side i.e. right side were comparable to that of non treated side i.e. Left side on all the days of sacrifice. This indicated that neither phosphate buffer saline (PBS) (50 mM, pH 7.2) nor the employed surgical procedure had any adverse effect on the reproductive vigour of the male mice. But when the mice were administered on both sides surgically, they were not able to survive. So, for the further studies mice were administered in one side only and the right side served as the test while left side as a control in all the experiments except in case of haematological parameters and serum level of testosterone, where PBS was administered in the right side of vas deferens of control mice. To check the effect of SIF, the animals were euthanized on day 3, 7, 14, 21, 30, 45, 60 and 90. The parameters evaluated include weight profile, TSI (%), seminal parameters, haematological parameters, serum level of testosterone, lipid peroxidation and histological changes.

### Weight profile and tissue somatic indices TSI (%)

Initial body weight of mice from each group was taken on the 1st day of the experiment and final weight on the last day of the experiment. For the estimation of TSI, mice were euthanized by cervical dislocation on the respective day of sacrifice. Mice were necropsied and various reproductive and non-reproductive organs were removed and weighed aseptically. The TSI (%) (Organ weight/body weight × 100) of reproductive (vas deferens, testes and cauda) and non-reproductive (Kidneys, liver, spleen and bladder) organs were estimated.

### Evaluation of sperm parameters

#### Total sperm count

Three mice from each group were sacrificed on the respective day of sacrifice. Immediately after necropsy, each vas deferens from both sidesi.e. right (inoculated) side and left (uninoculated) side was pulled out, placed in two glass plates containing freshly prepared, pre-warmed 200 μl of PBS (50 mM, pH 7.2). Mild teasing was done to enable the spermatozoa to swim out into the buffer. A fixed volume of 10 μl of the sample was placed on a glass slide, covered with a coverslip and examined under a light microscope at 400X magnification. Around eight to ten fields were scanned and the mean number of spermatozoa in all the fields were multiplied by 10^6^.

#### Sperm motility

Ten microliter of the sample was placed on a warm slide and examined (400X) under a light microscope. The motile and non-motile sperms were counted in eight to ten fields and the percentage of motile sperms was determined.

#### Hematological parameters

To estimate haematological parameters, mice were divided into two groups viz. control group (*n* = 6) and test group (n = 6) receiving 20 μl of PBS and 200 μg of SIF, respectively via Intra vas deferens route. Blood was collected on day 90 by cardiac puncture. For this, a 3 cc syringe with a 22–25 gauge × 1″ needle was used. The mouse was held by the scruff with the body hanging vertically. The heart was located by palpation of the heartbeat. The heart was punctured and slight back pressure was applied. Blood started to flow into the syringe automatically. Six hundred to eight hundred microliter of blood was collected and stored in the vial coated with anticoagulant and analysed for red blood cells (RBCs) and white blood cells (WBCs) counts, haemoglobin (Hb) concentration and the hematocrit serum collection, blood was collected in the vial (without anticoagulant coating) by the same procedure mentioned above and was analyzed to measure the activities of alanine aminotransferase (ALT) and aspartate aminotransferase (AST) according to the method of Reitman and Frankel [10].

#### Testosterone assay

Mice were divided into two groups viz. control and test consisting of 3 mice each. The control group was administered with 20 μl of PBS whereas the test group was administered with 200 μg of SIF via Intra vas deferens route. The serum was collected on day 90 as described above and the serum level of testosterone was measured by ELISA using a commercial kit as per manufacturer’s instructions. The sensitivity of the assay was 5 pg/ml with Intra and inter-assay coefficient of variations being 5.1 and 7.5%, respectively.

#### Lipid peroxidation

Mice were divided into two groups viz. control (PBS) and test (200 μg SIF) consisting of 6 mice each. They were sacrificed on day 3 and 7 and the level of lipid peroxidation was determined by the method of Ohkawa et al. [11]. For this, 3.3 ml TBA reagent (0.2 ml of 8.0% SDS, 1.5 ml of 20% acetic acid, 1.5 ml of 0.8% aqueous thiobarbituric acid and 0.1% of butylated hydroxyl toluene) was mixed with 0.2 ml tissue supernatant obtained after the homogenization of testes and the mixture was boiled at 95 °C in a water bath for 60 min. The solution was cooled and centrifuged at 2000 rpm for 10 min. The supernatant was then used for recording absorbance against blank (Distilled water) at 532 nm.

#### Histological analyses

Mice were sacrificed on the respective day of sacrifice and organs of mice were removed and fixed in 10% formaldehyde for 72 h. After 72 h, the tissues were embedded in paraffin. The paraffin tissue sections of 4 mm were stained with hematoxylin-eosin. Further, slides were observed at 400X magnification for any significant changes.

### Evaluation of SIF-binding receptor (SBR) from mouse spermatozoa as ameliorating agent against SIF-induced impairment of reproductive vigour

#### Extraction of spermatozoa from mice

The 6–7 week-old BALB/c male mice were sacrificed by cervical dislocation, and spermatozoa from the vas deferens were collected in prewarmed 50 mM PBS by gentle teasing and the count was set at 40 × 10^6^ spermatozoa/ml.

#### Isolation and purification of SIF binding receptor from mouse spermatozoa

Mouse spermatozoa-SIF binding receptor (MS-SBR) could be extracted by following a protocol already standardized in our laboratory by Thaper et al. [12]. For extraction, sperm suspension (10^8^ spermatozoa) was centrifuged and the pellet was washed twice with PBS before being subjected to ultrasonication (10 bursts of 15 s with 30s interval in between) using a probe sonicator. After centrifugation at 5000 rpm, both the cell pellet and sonicated supernatant were observed for the blockage of sperm immobilization. Since the blocking activity was found to reside in the pellet so it was subjected to different molarities of NaCl viz. 1,2,3 or 4 M, for different time intervals (2, 4, 8 and 12 h) at 37 °C under shaking conditions (220 rpm). The treated mixture was then centrifuged at 1500 rpm for 10 min and both pellet and supernatant were dialysed, concentrated and examined for blockage of SIF induced sperm immobilization and further purified on Sephadex G-200 column.

#### Evaluation of SIF binding receptor (SBR) as ameliorating agent

For this, male BALB/c mice were divided into 6 groups with 3 mice in each group. The administered volume for each mouse was 20 μl. Each mouse was intravasally administered with 200 μg of SIF pre-incubated for 30 min at 37 °C with Phosphate buffer saline PBS (Group I), 100 μg SBR (Group II), 200 μg SBR (Group III), 300 μg SBR (Group IV), 400 μg SBR (Group V) and 500 μg SBR (Group VI). All the mice from all the experimental groups were evaluated for sperm parameters.

## Results

### Isolation and purification of SIF from *S. aureus*

Isolation and purification of SIF was done by the method already standardized in our laboratory. Briefly, SIF was purified with ammonium sulphate precipitation followed by passing the bioactive fractions through Sephadex G-200 column and finally by DEAE-cellulose column (Suppl. Figs. 1, 2). Lastly, the molecular weight was estimated by SDS PAGE (Suppl. Fig. 3).

### Weight profile

Single unilateral administration of different concentrations of SIF revealed no statistically significant changes in the bodyweight profile of male mice in all the groups (Suppl. Fig. 4).

### Tissue somatic indices (TSI %)

The TSI of reproductive and non-reproductive organs excised from all the mice receiving different concentrations of SIF were determined on all the days of sacrifice and the results so obtained indicated no significant changes in the TSI of all the organs (Suppl. Fig. 5, 6).

### Evaluation of seminal parameters

To assess treatment-related changes in seminal parameters, mice (*n* = 3) were euthanized on the respective day of sacrifice and sperm count and motility were assessed and it was found that unilateral administration of SIF had sperm impairing effects on the side of treatment only whereas the left side showed no changes in seminal parameters. When the mice were treated with 10 μg of SIF, the right side displayed azoospermia (no spermatozoa) that persisted till day 7. On the other hand, left side displayed normal sperm count (17.3 ± 6.11 × 10^6^/ml). The sperm count started to restore thereafter, and on day 14, it raised to (10 ± 2.46 × 10^6^/ml) as compared to zero on day 7. This was, however, less by 37% in comparison to left side. Further, on day 21 the sperm count on right side became comparable to the left side. In case of motility, on day 3 and 7, motility could not be assessed due to azoospermia. On day 14, the motility on the right side was 16.3 ± 5.03% as compared to left side where motility was 33 ± 9.5%. By day 21, motility of the right side (33 ± 5.5%) became comparable to left side (32.6 ± 6.5%). When the mice were treated with higher concentration of SIF i.e. 50 μg, azoospermia persisted in the right side up to day 14, however, the left side showed normal sperm count (19 ± 3.60 × 10^6^/ml). Thereafter, the sperm count started to restore and on day 21, it raised to 14.6 ± 3.21 × 10^6^/ml as compared to zero on day 14. This was, however, less by 33% as compared to left side. Further, on day 30, the sperm count in the right side (18.3 ± 0.51 × 10^6^/ml) became comparable to the left side (19 ± 3.60 × 10^6^/ml). In case of motility, till day 14, motility could not be assessed due to azoospermia in the right side. On day 21, the motility on the right side was 19 ± 1% as compared to left side where motility was found to be 37 ± 8.7%. By day 30, motility of the right side (41.6 ± 12.05%) became comparable to left side (42.6 ± 9.7%). Mice treated with 100 μg of SIF showed azoospermia in the right side till day 30 with respect to left side which showed normal sperm count (26.6 ± 12.08 × 10^6^/ml). The sperm count started to restore thereafter and on day 45 the sperm count of right side (24.6 ± 5.03 × 10^6^/ml) was comparable to the left side (24 ± 5 × 10^6^/ml). Motility could not be assessed till day 30 due to complete azoospermia in the right side. By day 45, motility of the right side (35.6 ± 5.68%) became comparable to left side (34.3 ± 7.02%). With the concentration, 200 μg of SIF, azoospermia could be achieved till day 90 in the right side whereas the left side displayed normal sperm count. Further, on all the days, the motility could not be assessed due to azoospermia in the right side (Table 1).

**Table 1:** Seminal parameters of male mice after intravasal administration of SIF

Conc. of SIF (μg)	Days																															
	Day 3				Day 7				Day 14				Day 21				Day 30				Day 45				Day 60				Day 90			
	Sperm count (× 10^**6**^)		Motility (%)		Sperm count (× 10^**6**^)		Motility (%)		Sperm count (× 10^**6**^)		Motility (%)		Sperm count (× 10^**6**^)		Motility (%)		Sperm count (× 10^**6**^)		Motility (%)		Sperm count (× 10^**6**^)		Motility (%)		Sperm count (× 10^**6**^)		Motility (%)		Sperm count (× 10^**6**^)		Motility (%)	
	L	R	L	R	L	R	L	R	L	R	L	R	L	R	L	R	L	R	L	R	L	R	L	R	L	R	L	R	L	R	L	R
**10**	**7**	**0**	**24±** **7**	**0**	**17.3 ± 6.11**	**0**	**22.3±** **5.68**	**0**	**16.6±** **1.52**	**10±** **2.46**	**33±** **9.5**	**16.3±** **5.03**	**26.3±** **13.31**	**26.6±** **12.22**	**32.6±** **6.5**	**33±** **5.5**	**ND**	**ND**	**ND**	**ND**	**ND**	**ND**	**ND**	**ND**	**ND**	**ND**	**ND**	**ND**	**ND**	**ND**	**ND**	**ND**
**50**	**18.33±** **4.04**	**0**	**44±** **7.5**	**0**	**22.6±** **6.50**	**0**	**55.6±** **5.03**	**0**	**19±** **3.60**	**0**	**24.3±** **6.50**	**0**	**21±** **6.24**	**14.6±** **3.21**	**37±** **8.7**	**19±** **1**	**19±** **3.60**	**18.3±** **0.51**	**42.6±** **9.7**	**41.6±** **12.05**	**ND**	**ND**	**ND**	**ND**	**ND**	**ND**	**ND**	**ND**	**ND**	**ND**	**ND**	**ND**
**100**	**19.6±** **4.04**	**0**	**26±** **9.1**	**0**	**17.6±** **5.68**	**0**	**22.66±** **5.68**	**0**	**21.6±** **6.02**	**0**	**25±** **6.5**	**0**	**18.6±** **5.03**	**0**	**30±** **7.2**	**0**	**26.6±** **12.08**	**0**	**30±** **7.5**	**0**	**24±** **5**	**24.6±** **5.03**	**34.3±** **7.02**	**25.6±** **5.68**	**ND**	**ND**	**ND**	**ND**	**ND**	**ND**	**ND**	**ND**
**200**	**15.6±** **4.72**	**0**	**15.6±** **3.51**	**0**	**21.3±** **5.03**	**0**	**24.6±** **8.08**	**0**	**18.6±** **4.50**	**0**	**25.6±** **7.50**	**0**	**20.3±** **4.16**	**0**	**19.3±** **9.07**	**0**	**19.3±** **3.78**	**0**	**22.6±** **5.03**	**0**	**23±** **4.35**	**0**	**33.6±** **4.50**	**0**	**18.6±** **6.02**	**0**	**26±** **8**	**0**	**21.3±** **5.03**	**0**	**26.6±** **4.50**	**0**

Data presented as mean ± SD; *L* Left side, *R* Right side, *ND* Not determined

### Hematological parameters

No significant changes were found in the levels of AST and ALT in serum and in other haematological parameters (Haemoglobin, RBC, WBC, haematocrit) in the test (SIF 200 μg) as compared to the control (PBS) throughout the course of the investigation (Table 2).

**Table 2:** Effect of intravasal administration of SIF (200 μg) on hematological parameters of male mice (values in mean ± SD)

Hematological parameters		
Parameters	Control (PBS)	SIF (200 μg)
**RBC (10**^**6**^**/mm**^**3**^**)**	4.87 ± 0.19	4.96 ± 0.15
**WBC (10**^**3**^**/mm**^**3**^**)**	5.67 ± 0.29	5.14 ± 0.26
**Hb (g/dl)**	14.2 ± 0.03	14.7 ± 0.02
**Lymphocytes (%)**	84.18 ± 0.01	85.24 ± 0.05
**Monocytes (%)**	2 ± 0.23	4 ± 0.31
**Neutrophils (%)**	12.7 ± 0.12	11 ± 0.21
**Eosinophil (%)**	0.06 ± 0.20	0.05 ± 0.32
**Basophil (%)**	0.09 ± 0.17	0.07 ± 0.23
**AST (u/ml)**	34.02 ± 1.09	31.68 ± 2.38
**ALT (u/ml)**	24.94 ± 0.36	25.31 ± 0.71
**Testosterone (ng/ml)**	1.72 ± 0.08	0.72 ± 0.05

### Testosterone assay

A significant decrease was noted in the serum testosterone level after intravasal administration of SIF (200 μg) in the test (0.72 ± 0.05, (*p* < 0.01)) as compared to the control (1.72 ± 0.08) receiving PBS (Table 2).

### Lipid peroxidation

**Fig. 1:**
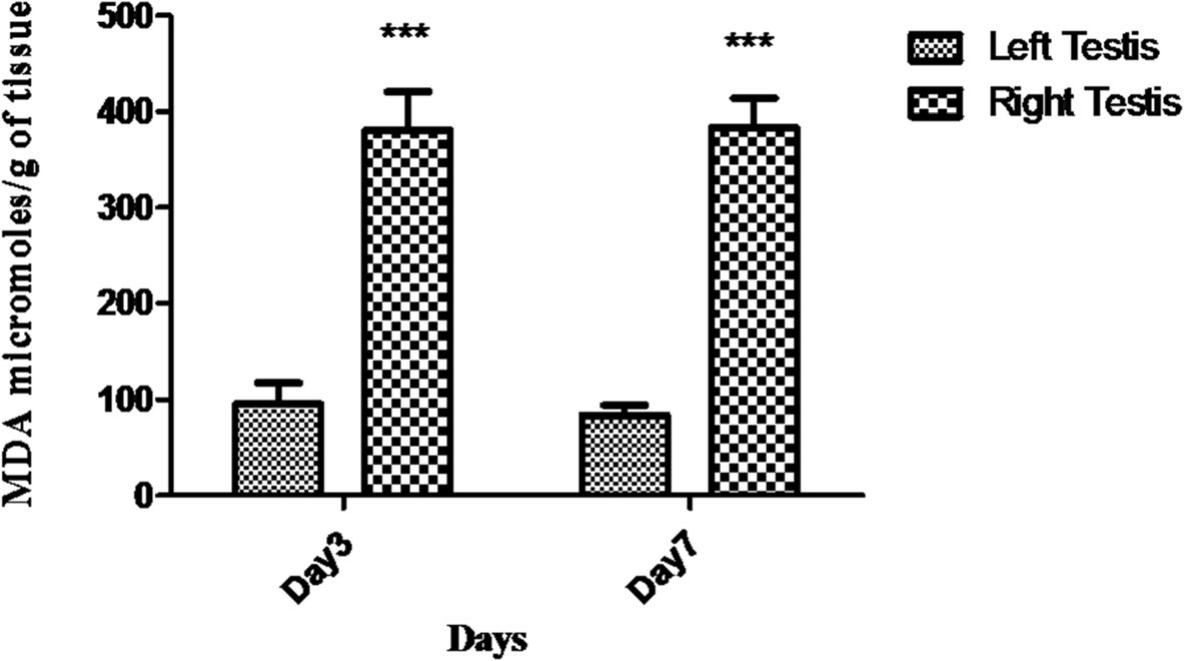
Lipid peroxidation in terms of MDA (μmoles/g of tissue) of testis in the right side in comparison to the left side on day 3 and 7. Data represents mean values± SD. (*), (**), (***) represent *p* < 0.05, *p* < 0.01, *p* < 0.001, respectively

Following the single unilateral administration of 200 μg SIF, a significant increase in endogenous thiobarbituric acid reactive substances (TBARS) levels was observed in the right side (372.74 μmoles/g of tissue and 381.94 μmoles/g of tissue) as compared to the left side (87.36 μmoles/g of tissue and 68. 95 μmoles/g of tissue) on day 3 and 7 (Fig. 1).

### Histological examination

**Fig. 2:**
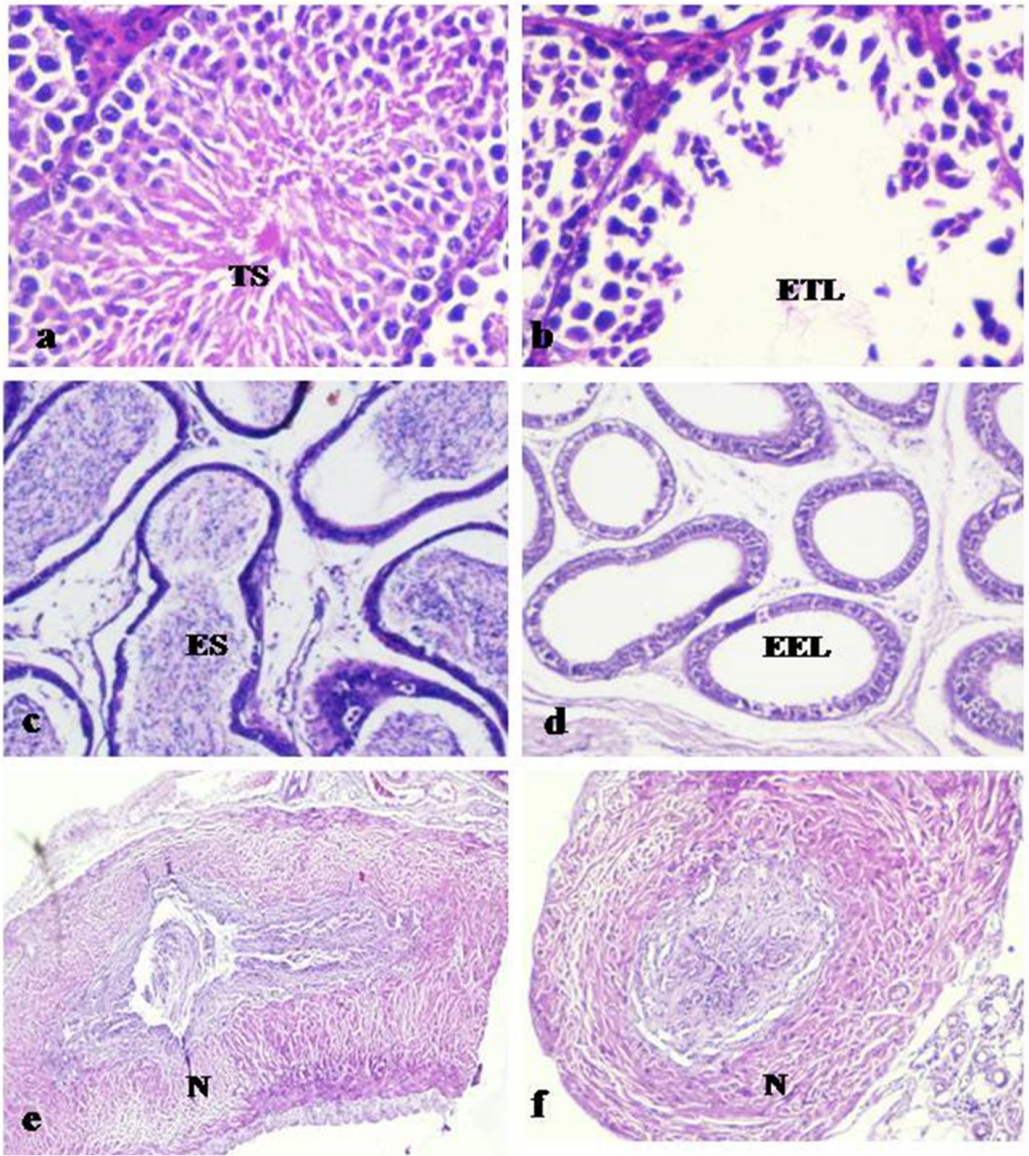
Representative photomicrographs of histological examination (day 90) of reproductive organs viz. testis, cauda epididymis and vas deferens of left side (**a**, **c**, **e**) and right side (**b**, **d**, **f**) receiving 200 μg SIF**.** Left testis- **a** Normal seminiferous tubule showing testicular spermatozoa (abbreviated as TS,100X). Left cauda epididymis- **c** Tubules filled with epididymal spermatozoa (abbreviated as ES,100X). Left vas deferens- **e** Normal tissue histology (abbreviated as N, 100X). Right testis- **b** Degenerative changes in the seminiferous tubules showing empty testicular lumen (abbreviated as ETL,100X). Right cauda epididymis- **d** Empty epididymal lumen (abbreviated as EEL,100X). Right vas deferens- **f** Normal tissue histology (abbreviated as N, 100X)

**Fig. 3:**
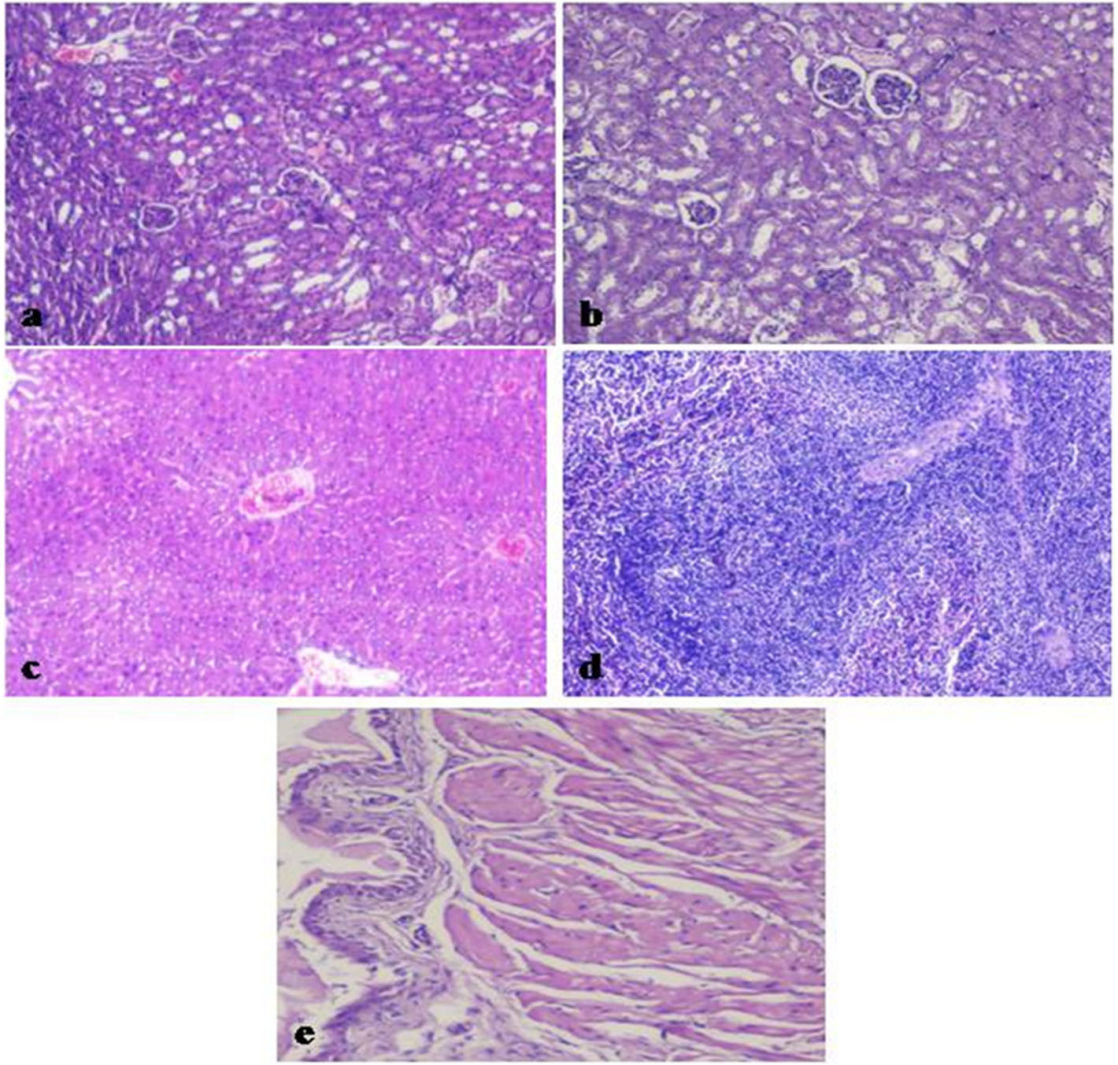
Representative histological photomicrographs (day 90) of various non-reproductive organs of male mice receiving 200 μg of SIF. **a** Left Kidney. **b** Right Kidney. **c** Liver. **d** Spleen and **e** Bladder showing normal histology

To check any adverse effect of SIF on tissues morphology of various reproductive (testis, cauda epididymis and vas deferens) and non-reproductive organs (spleen, kidney, bladder and liver), histological analysis was carried out. The right set of organs of mice treated with SIF (200 μg) showed azoospermia with regressive changes viz. loosening and sloughing of germ cells in testes (Fig. 2b), caudal epididymis showed empty tubules (Fig. 2d), while vas deferens showed normal tissue histology (Fig. 2f). However, the left set of reproductive organs revealed normal tissue histology i.e. testes showed regular seminiferous tubule and germinal cell morphology (Fig. 2a), epididymis displayed the well-vascularized loose connective tissue present around the epididymal duct (Fig. 2c) and vas deferens showed normal columnar epithelium (Fig. 2e). While all the non-reproductive organs were found to be histologically normal in all the cases (Fig. 3).

### Evaluation of SIF-binding receptor from mouse spermatozoa as ameliorating agent against SIF-induced impairment of reproductive vigour

#### Isolation and purification of SIF binding receptor from mouse spermatozoa

The receptor was isolated and purified by Deepali Thaper [12]. Briefly, SIF binding receptor on surface of mouse spermatozoa was extracted by treating the sperm pellet with 4 M NaCl for 2 h at 37 °C and further purified by molecular sieve chromatography.

#### Evaluation of SIF binding receptor (SBR) as ameliorating agent

In order to assess the amelioration of SIF induced changes in reproductive vigour of male mice, mice were administered with SIF pre-incubated with PBS/SBR by intra vas deferens route and 3 mice from each group were sacrificed at 72 h post treatment and seminal parameters were evaluated.

#### Sperm parameters

The sperm count in right vas deferens of mice administered with SIF pre-incubated with PBS or lower concentrations (100 and 200μg) of SBR showed azoospermia in comparison to left vas deferens. However, test Group IV, in which SIF pre-incubated with 300 μg SBR was administered, restoration of 67% of the sperm count occurred in the right vas deferens in comparison to left vas deferens while Group V (400 μg) and VI (500 μg) showed no change in the sperm count of right side in comparison to the left side (Table 3).

**Table 3:** Sperm parameters of male mice after 72 h of treatment with 200μg of SIF and different concentrations of SBR

Sperm parameters	Group I (PBS)		Group II (100 μg)		Group III (200 μg)		Group IV (300 μg)		Group V (400 μg)		Group VI (500 μg)	
	L	R	L	R	L	R	L	R	L	Ri	L	R
**Sperm count (× 10**^**6**^ **/ml)**	37 ± 3.13	0	29 ± 4.03	0	32 ± 3.16	0	29 ± 1.07	19 ± 2.01	37 ± 1.71	39 ± 3.15	27 ± 2.08	26 ± 1.15
**Motility (%)**	54 ± 2.01	0	41 ± 1.07	0	47 ± 5.21	0	31 ± 5.08	9 ± 3.12	52 ± 4.15	49 ± 3.17	48 ± 2.17	47 ± 3.11

Data presented as mean ± SD; *L* Left side, *R* Right side

Since, azoospermia was observed, in case of Group I, II and III, hence, motility could not be assessed for the same. In case of test Group IV i.e. those administered with 300 μg SBR, there was 71% decrease observed in the right side in comparison to the left side and in case of Group V and VI, the motility in the right side was comparable to the left side (Table 3).

## Discussion

Previously in our laboratory, strain of *S. aureus* was found to produce microbial factor with sperm immobilizing property in-vitro. Moreover, it was also found to possess excellent contraceptive efficacy in female BALB/c mice following intravaginal administration [9]. The encouraging findings obtained with this microbial factor in the female mice have led to resurgence of interest in exploitation of the same as a potential candidate for male contraception.

Hence, the present study was aimed at evaluating the effect of the sperm immobilization factor (SIF) from *S. aureus* on reproductive potential of male mice via vas deferens route of administration. To explore this possibility, the effect of SIF on body weight, reproductive and non-reproductive organ weight, seminal parameters, haematological parameters, testosterone assay, lipid peroxidation and tissue histology was examined at four different doses viz. 10, 50, 100 or 200 μg for each mouse for 90 days.

The body and organ (reproductive and non reproductive) weight profile of male mice showed absence of any significant change indicating the general well being of the animals*.* The results obtained in the present study are in line with the studies conducted previously with various antifertility compounds in different animal models [8, 13–17].

The results of seminal parameters revealed that 200 μg of SIF was able to cause azoospermia up to 90 days. These results are in corroboration with Upadhyay et al. [8] who have shown that single unilateral administration of neem oil in male rats led to azoospermia in the side of treatment only. The disturbance in seminal parameters of the treatment side, as observed in the present study, was further supported by the findings of histological examination of reproductive organs, the results of which showed that in comparison to left side wherein normal histology was observed, right side showed severe histological changes as evidenced by empty lumens in right testis and caudal epididymis. This is suggestive of loss of competence of seminiferous epithelium to produce germ cells in testes. The most likely explanation for this severe degeneration could be the probable loss of germ cells and damage to testicular somatic cells. Various studies in the past have reported similar findings with other antifertility agents viz. gossypol tetra acetic acid [18] and the leaf extracts of *Azadirachta indica* [19], *Allamanda cathartica* [20], *Curcuma longa* [21], *Dalbergia sissoo* [22], and SC12937 [23].

Several studies have demonstrated that agents with potent contraceptive effect must do so without affecting the haematological parameters e.g. Verma and Singh, [24] (*Coccinia indica*), Sharma and Jocob, [25] (*Mentha arvensis*) and Etim et al. [26] (*Achyranthes aspera*). Following the similar course, it seemed relevant to perform the haematological tests, serum level of ALT and AST which form the very front-line investigations on which diagnosis of various diseases is based as they provide vital information about general health and metabolism of the individuals. Unaltered haematological parameters in all of the test groups in the present investigation in mice suggest that SIF did not cause any adverse effects on the general health of the animals. Further, the serum levels of ALT and AST were also found to fall in normal range in all the test groups. Thus, these observations suggest that SIF neither induces any systemic toxicity nor does it have an adverse effect on the general metabolism of the body. This contention is also supported by absence of histological changes in the non reproductive organs of SIF treated mice in all the experimental groups.

It is a well-known fact that spermatogenesis is a testosterone dependent process [27] and deprivation of the same suppresses the genesis of spermatozoa [28]. When the testosterone levels were evaluated in the present study, the decreased levels in the test group, as compared to the control group, suggested that the factor-mediated suppression of testosterone concentrations might be attributed to degeneration of leydig cells. However, it is important to state here that since hypospermatogenesis was observed only in the right side, hence, degeneration of Leydig cells could not be considered as the sole reason for the hypospermatogenesis. As a result, it could be anticipated that some other factor might also be contributing to this.

To gain an insight into the other possible mechanism by which SIF might have affected the spermatogenesis oxidative stress was evaluated. As, it has been suggested to affect fertility negatively via inflammation (measured in terms of MDA levels) [29]. Hence, when levels of MDA in tissue homogenates of testes were estimated using TBARS assay, the results indicated significantly high levels of MDA in right testis administered with SIF in comparison to left testis. This rise in the level of MDA in the treated testis implies that the oxidative stress conditions in the treated side might have also played a crucial role in inhibiting the spermatogenesis and steroidogenesis.

Further, to ratify SIF induced azoospermia, another study was carried out where male mice were administered with SIF pre incubated with SIF binding receptor isolated from mouse spermatozoa via intra vas deferens route. For this, SIF (200 μg) pre-incubated with PBS or different concentrations of SBR were given in the right vas deferens and the left vas deferens served as control. When SIF pre-incubated with PBS or SBR was administered, the sperm count in right side displayed a complete absence of spermatozoa in comparison to the left side in group I, II and III, while group IV showed restoration of sperm count in the right side in comparison to the left side. However, group V and VI showed comparable sperm count on both sides. Therefore, from the aforementioned results, it can be concluded that SIF is able to compromise the reproductive function of mice which can be ameliorated by preincubation with SIF binding receptor. Earlier also it has been observed that SBR causes blockage of SIF induced contraception when administered in female mice again, substantiating the contraceptive efficacy of SIF [12]. Thus, from the present study, it can be concluded that SIF causes loss of male reproductive competence, when administered via intra vas deferens route and SIF binding receptor isolated from mouse spermatozoa was able to ameliorate the deleterious effect of SIF on male reproductive vigour. In future immunofluorescence assay or the tunnel assay and western blot will be carried out to better understand the underlying mechanisms at the molecular level involved behind the effect of SIF.

## Conclusion

SIF via the intravasal administration at a dose of 200 μg causes azoospermia up to 90 days without eliciting detectable toxic effects.

## Supplementary information

**Additional file 1 **Suppl. Fig. 1: Elution pattern of SIF from *S. aureus* after gel filtration through Sephadex G-200 column showing the presence of sperm immobilization activity in fractions 4–5 with a peak value in fraction 4. Suppl. Figure 2: Elution pattern of SIF from *S. aureus* obtained after DEAE cellulose column showing sperm immobilization activity in fractions 14–16 with peak value in fraction 15. Suppl. Figure 3: Estimation of molecular weight of purified SIF by SDS-PAGE; a) Lane 1- Marker, b) Lane 2- SIF. **Suppl. Fig. 4**: Body weight response of male Balb/c mice administered intravasally with SIF a) 10 μg b) 50 μg c) 100 μg d) 200 μg. **Suppl. Fig. 5**: Tissue somatic indices (%) of various reproductive organs of mice administered intravasally with SIF a) 10 μg b) 50 μg c) 100 μg d) 200 μg. **Suppl. Fig. 6**: Tissue somatic indices (%) of various non reproductive organs of mice administered intravasally with SIF a) 10 μg b) 50 μg c) 100 μg d) 200 μg

## Data Availability

All data generated or analyzed during this study are included in this article.

## Abbreviations

SIF: Sperm immobilization factor
TSI: Tissue somatic indices
PBS: Phosphate buffered saline
PEG: Polyethylene glycol
SDS PAGE: Sodium dodecyl sulfate polyacrylamide gel electrophoresis
