# Supplementary material for: Sperm impairing microbial factor: potential candidate for male contraception
Source: Reprod Biol Endocrinol. 2020 Sep 30;18:96. doi: 10.1186/s12958-020-00654-4 (PMC7526221; doi:10.1186/s12958-020-00654-4)
Supplement: Supplementary file 1 — Additional file 1 Suppl. Fig. 1: Elution pattern of SIF from S. aureus after gel filtration through Sephadex G-200 column showing the presence of sperm immobilization activity in fractions 4–5 with a peak value in fraction 4. Suppl. Figure 2: Elution pattern of SIF from S. aureus obtained after DEAE cellulose column showing sperm immobilization activity in fractions 14–16 with peak value in fraction 15. Suppl. Figure 3: Estimation of molecular weight of purified SIF by SDS-PAGE; a) Lane 1- Marker, b) Lane 2- SIF. Suppl. Fig. 4: Body weight response of male Balb/c mice administered intravasally with SIF a) 10 μg b) 50 μg c) 100 μg d) 200 μg. Suppl. Fig. 5: Tissue somatic indices (%) of various reproductive organs of mice administered intravasally with SIF a) 10 μg b) 50 μg c) 100 μg d) 200 μg. Suppl. Fig. 6: Tissue somatic indices (%) of various non reproductive organs of mice administered intravasally with SIF a) 10 μg b) 50 μg c) 100 μg d) 200 μg [file 12958_2020_654_MOESM1_ESM.docx]

**
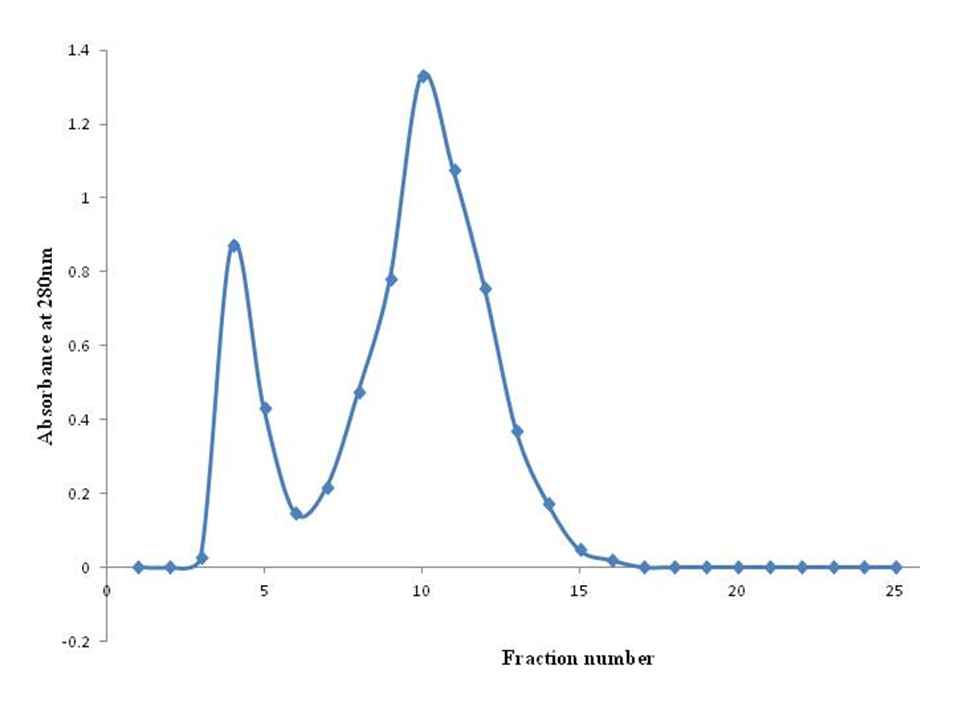
**

**Suppl. Fig. 1: Elution pattern of SIF from *S. aureus* after gel filtration through Sephadex G-200 column showing the presence of sperm immobilization activity in fractions 4-5 with a peak value in fraction 4**

**
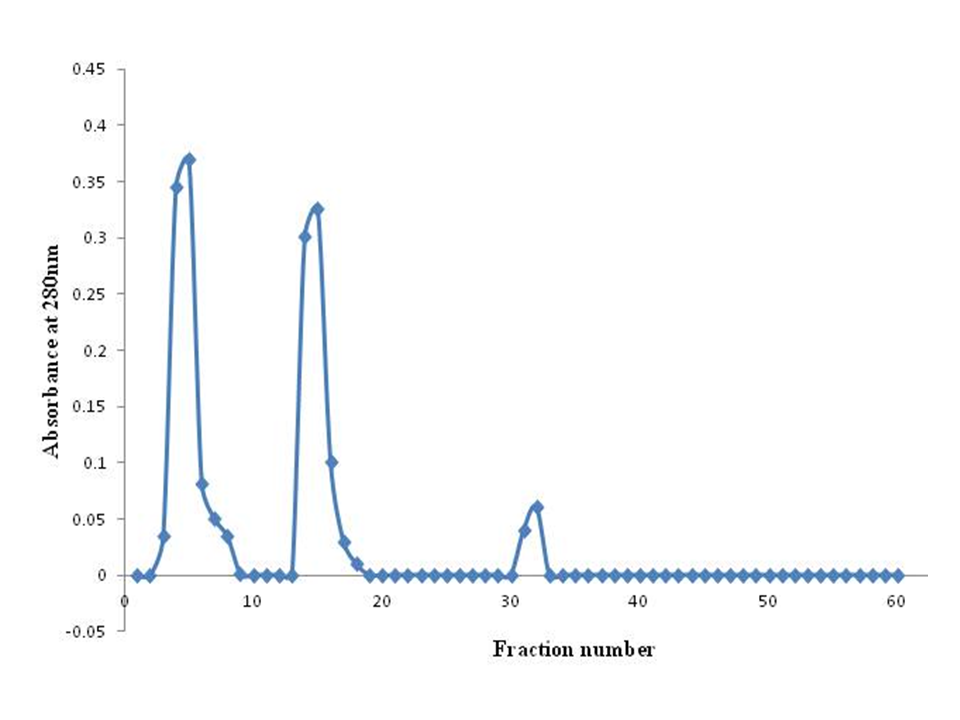
**

**Suppl. Figure 2: Elution pattern of SIF from *S. aureus* obtained after DEAE cellulose column showing sperm immobilization activity in fractions 14-16 with peak value in fraction 15**

**
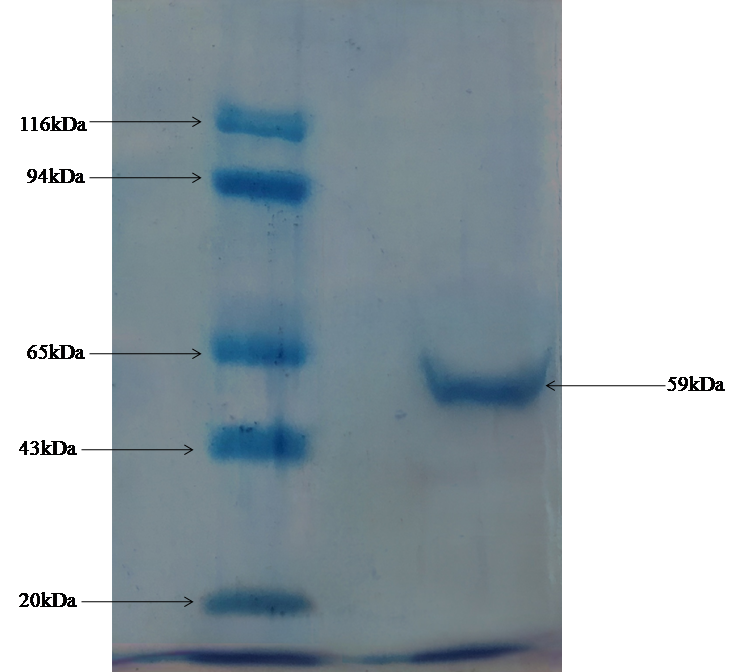
**

**Suppl. Figure 3: Estimation of molecular weight of purified SIF by SDS-PAGE; a) Lane 1- Marker, b) Lane 2- SIF**

**
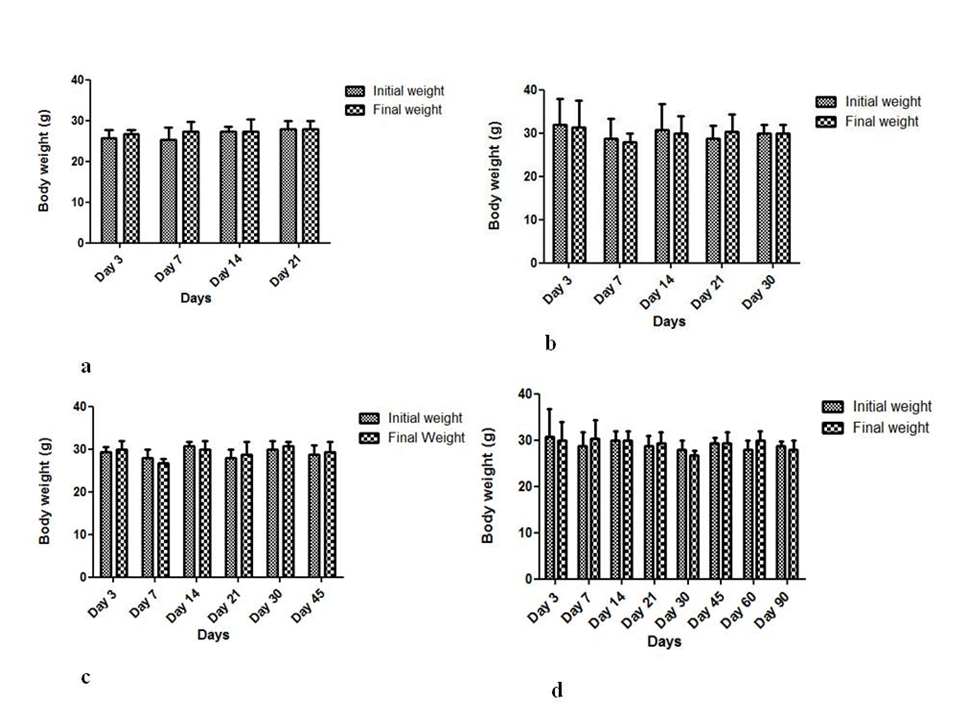
**

**Suppl. Fig. 4: Body weight response of male Balb/c mice administered intravasally with SIF a) 10 μg b) 50 μg c) 100 μg d) 200 μg**

**
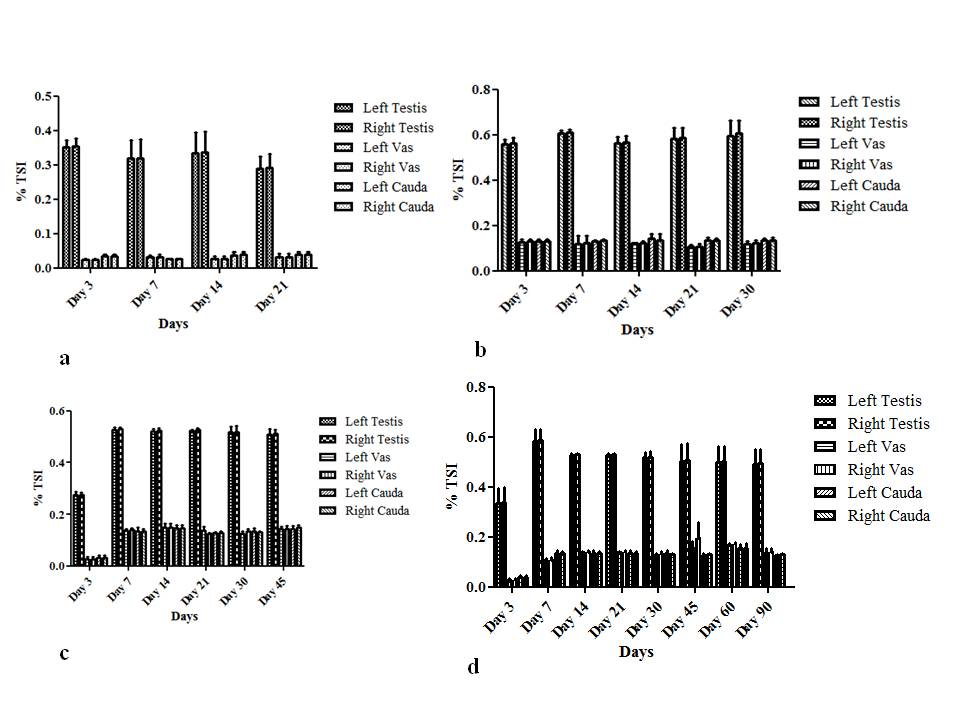
**

**Suppl. Fig. 5: Tissue somatic indices (%) of various reproductive organs of mice administered intravasally with SIF a) 10 μg b) 50 μg c) 100 μg d) 200 μg**

**
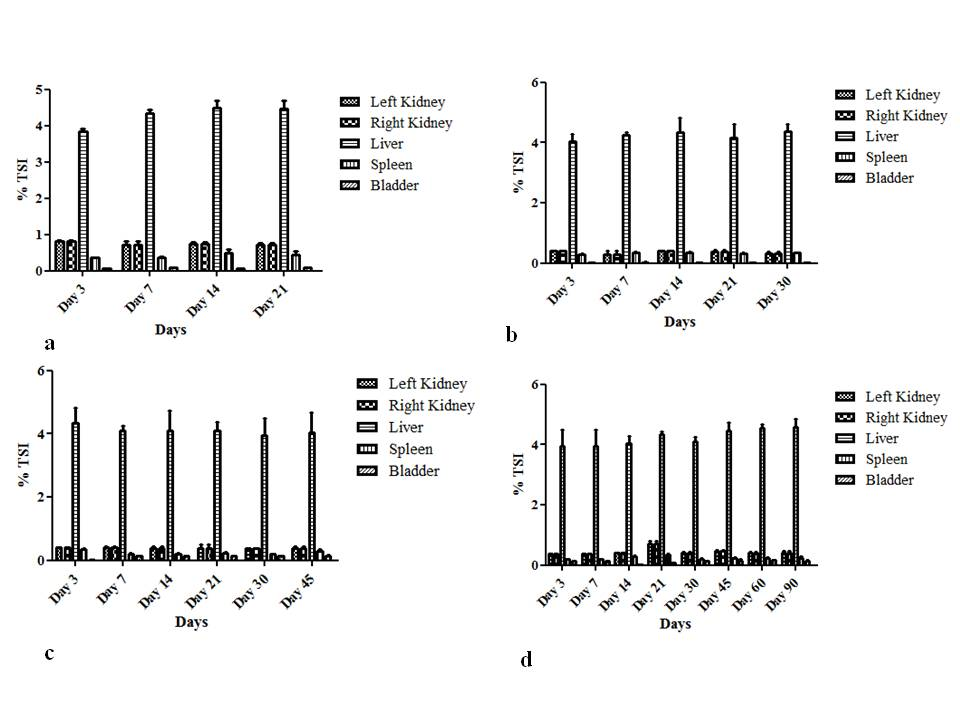
**

**Suppl. Fig. 6: Tissue somatic indices (%) of various non reproductive organs of mice administered intravasally with SIF a) 10 μg b) 50 μg c) 100 μg d) 200 μg**
